# *Candida glabrata* oropharyngeal infection in a patient with oral squamous cell carcinoma after COVID-19 infection

**DOI:** 10.22034/cmm.2023.345120.1478

**Published:** 2023-09

**Authors:** Jalal Jafarzadeh, Javad Javidnia, Seyed Ali Jeddi, Mahshid Vakili, Mojtaba Taghizadeh Armaki, Mahin Tavakoli

**Affiliations:** 1 Department of Medical Mycology and Parasitology, School of Medicine, Babol University of Medical Sciences, Babol, Iran; 2 Invasive Fungi Research Center, Communicable Diseases Institute, Mazandaran University of Medical Sciences, Sari, Iran; 3 Department of Laboratory Sciences, School of Allied Sciences, Abadan University of Medical Sciences, Abadan, Iran; 4 Department of Bacteriology and Virology, School of Medicine, Semnan University of Medical Sciences, Semnan, Iran; 5 Infectious Diseases and Tropical Medicine Research Center, Health Research Institute, Babol University of Medical Sciences, Babol, Iran; 6 Departments of Medical Mycology, Faculty of Medical Sciences, Tarbiat Modares University, Tehran, Iran

**Keywords:** *Candida glabrata*, COVID-19, Oropharyngeal candidiasis, Squamous cell carcinoma

## Abstract

**Background and Purpose:**

The COVID-19 pandemic may be an aggravating risk factor for the delay of the diagnoses of serious illnesses, such as oral squamous cell carcinoma, as well as poor management of patients with underlying morbidities, the onset of oral lesions, and antifungal susceptibility to opportunistic fungal infections. Oral candidiasis is one of the most common oral features of COVID-19.

**Case Report:**

This study aimed to report an 83-year-old female diagnosed with oral carcinoma who developed oropharyngeal candidiasis after falling ill with COVID-19. In late 2020, this patient was hospitalized for COVID-19 pneumonia. A fissured tongue with white scars appeared after the COVID-19 recovery that caused pain, dysphasia, and dysarthria. The sequencing result based on the internal transcribed spacer rDNA region confirmed *Candida glabrata*. Its antifungal susceptibility showed susceptibility to nystatin, fluconazole, and caspofungin, but resistance to the other azoles and amphotericin B.

**Conclusion:**

Risk of fungal infections, such as *Candida* seems to be high in patients with severe COVID-19, mainly affecting the oral mucosa. However, whether they are directly attributed to COVID-19 or other surrounding factors is unknown.

## Introduction

The coronavirus disease 2019 (COVID-19) pandemic has posed a serious burden on the global healthcare system in both developed and developing nations and caused a great number of deaths [ 1 ]. Fungal co-infections or superinfections may cause poor clinical outcomes and mortality due to COVID-19 in older people and immunocompromised patients [ 1 ]. Oral lesions are commonly found as COVID-19 oral manifestations which are influenced by various factors, such as undefined pharmacological treatment for COVID‐19, underlying diseases, invasive therapeutic methods, and medication [ 2 ]. 

Buccal mucosa and tongue are reported to be the most frequent locations of oral candidiasis that can be disseminated to the esophagus (oropharyngeal candidiasis) particularly in immunocompromised patients [ 3 ]. Oropharyngeal candidiasis (OPC) is an important problem in 25% of patients with head and neck cancer that can result in pain, dysphasia, malnutrition, and esophageal or systemic infection [ 4 ]. *Candida albicans* is reported as the most frequent colonizer in the oral cavity which significantly predisposed immunocompromised patients to OPC [ 5 ]. Extensive use of corticosteroids in COVID-19 patients may also continue to increase the risk of COVID-19-related *Candida* infections. 

This study aimed to present a case of *Candida glabrata* OPC infection in a patient with oral squamous cell carcinoma after infection with COVID-19.

## Case Report

**Figure 1:**
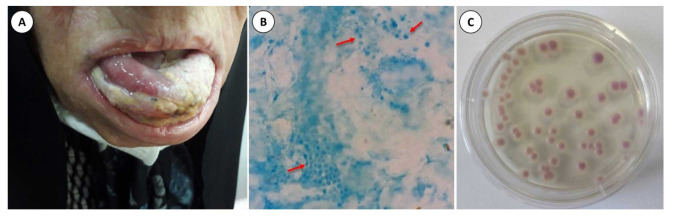
**A.** Intraoral photograph showing diffuse swelling along with the white and yellow lesions that appeared on the lateral and dorsal surfaces and the tip of the tongue **B.** Methylene blue staining of the sample from tongue lesions showed the presence of yeasts (X40). **C.** Growth of pink/purple colonies of *Candida glabrata* on CHROMagar *Candida* medium after 3 days of incubation at 35 °C.

In July 2019, an 83-year-old female with oral squamous cell carcinoma presented to a Medical Center in Babol, Iran, and received radiation treatment twice a month. In June 2020, during the second wave of the COVID-19 pandemic, this case was admitted to the hospital with a diagnosis of COVID-19 pneumonia. After the COVID-19 recovery, the tongue appeared white and yellow; therefore, this patient was referred to Babol University-based medical science laboratory setting (Figure 1).

A tongue dorsum swab was examined with hydrogen peroxide. Afterward, this sample was detected as *C. glabrata* with culturing on CHROMagar^TM^ *Candida* (CHROMagar *Candida*, France) medium and confirmed with sequencing internal transcribed spacer region [ 6 ]. The sequence was submitted to the GenBank database under the accession number OR484916.1. 

Subsequently, this patient was treated with fluconazole three times per day, and topical antifungal miconazole four times per day, and her symptoms started to improve within 3 weeks of starting antifungals. Furthermore, the *in vitro* activities of 10 antifungals, including caspofungin, nystatin, amphotericin B, tioconazole, voriconazole, itraconazole, econazole, ketoconazole, clotrimazole, and fluconazole against this species was studied according to the Clinical and Laboratory Standard Institute (M27-A3/S4) broth microdilution document [ 7 ].

*Candida glabrata* showed low activity against tioconazole, amphotericin B, clotrimazole, voriconazole, itraconazole, econazole, ketoconazole, with minimum inhibitory concentration (MIC) values of 64, 32, 8, 8, 4, 4, and 4 µg/ml, respectively. However, this species was susceptible to fluconazole, nystatin, and caspofungin with MIC values of 8, 0.5, and 0.25, respectively.

## Discussion

Emergence and expansion of COVID-19 caused a potential threat to global public health [ 8 ]. Patients with underlying health conditions, such as cancer are at greater risk of serious COVID-19 infection [ 9 ]. In addition, a higher incidence rate of fungal co-infections or superinfection was reported in patients with COVID-19 with rates of 23.3%, 2.5%, 0.8%, and 0.4% for *Aspergillus*, *Mucor*, *Candida*, and *Cryptococcus infections*, respectively [ 10 ]. 

Severity of COVID-19 infection can lead to *Candida* colonization/infection and also qualitative shifts towards a higher identification rate of non-*albicans Candida* species which is probably due to immunodepression, increasing antimicrobial resistance, and extensive antimicrobial use [ 11 ]. In addition to being more vulnerable to COVID-19 and immune dysregulation caused by corticosteroids, the higher severity of OPC is also associated with underlying disease and lower immunity among the elderly [ 12 ]. 

To date, *C. albicans* is also responsible for the majority of OPC episodes in patients with head and neck cancer, and *C. glabrata* ranks as the second or third most common cause of OPC after *C. albicans* [ 13 ]. It was noted that *C. glabrata* is usually isolated along with other *Candida* species, compared to other non-*albicans Candida*, and single-species infection due to this species alone is rare [ 14 ]. Meanwhile, *C. glabrata* may be emerging as a potential pathogen in elderly populations, for example, its oropharyngeal colonization has been reported in 29% of adults over 80 years of age [ 12 ]. The greatest risk factor for the development of breakthrough infections caused by C. glabrata is a high resistance rate to azoles, particularly fluconazole; however, the isolate recovered from the present case was susceptible to fluconazole [ 15 ]. Overall, COVID-19 infection puts patients at risk of developing OPC; therefore, these patients need to undergo an oral cavity assessment to ascertain the presence of OPC.

## Conclusion

Older adults with underlying morbidities are at a significantly increased risk of severe diseases following infection from COVID-19. Subsequently, the management of OPC is essential for early diagnosis and proper identification of the pathogen. Moreover, its antifungal susceptibility testing is crucial for the selection of the appropriate therapeutic strategies.
